# Supplementary material for: Barriers and facilitators to hepatitis C patient engagement: Interview study with general practitioner champions in England
Source: PLoS One. 2026 Jul 10;21(7):e0337867. doi: 10.1371/journal.pone.0337867 (PMC13353960; doi:10.1371/journal.pone.0337867)
Supplement: S1 File — (PDF) [file pone.0337867.s001.pdf]

S1 Appendix - Topic Guide

General introduction, information and consent confirmation, etc

Setting the scene:

Since the 'new' direct acting antiviral treatment for Hep C (2017) most of the people who've now been treated were found through drug services, Hep C Trust peers, and prison services. Now the focus is moving towards identifying people LTFU who were diagnosed some time ago, before advent of new drugs, as well as identifying people in community who may not know they're at risk – primary care is likely to be a main point of contact for these people.

DEFINE RE-ENGAGEMENT – people lost to follow up who may have had some experience of treatment, or who may only ever have had a hep c antibody result some time ago.

[in brackets – the COM-B domain the question belongs to]

INTERVIEW VERSION

About GPs and their role in HCV care (warm up questions 5-10 minutes)

1. Can you please describe your current practice, and how long you have been in this role? [motivation/ professional role and identity]
2. What region of England is this in?
3. To what extent, in your GP practice, have you acquired knowledge and skills of Hepatitis C? [psychological capability/knowledge/skills]
   - How confident do you feel in Hep C?
4. In general, is guidance or training on HCV available to GPs? [capability/knowledge/skills]
   - Prompts: How could training be improved? How much awareness is there of different patient needs and newer treatments?
5. What is your current involvement in Hep C care (in general)?

   Prompts: flagging patients with risk factors, using the PSI Tool, testing, referral for treatment, SVR testing after treatment? Contacting people lost to follow up?

6. Regarding Hep C care, do you communicate or liaise with other services? [opportunity-social]
   - Prompts: ODNs [assume yes if they are a GP champion], drug & alcohol services, prison services, community outreach, peers?
   - Prompts: what is working well? what is not working well?
7. Are you aware of the new Hepatitis C dashboard produced by UKHSA?

Re-engagement of historically diagnosed patients

The next questions are focused on the re-engagement of patients who have been lost to follow up. It may not be relevant to your current work but we would appreciate you sharing your thoughts.

8. What are you currently doing for the re-engagement of patients lost to follow up? [motivation/social/professional role and identity, 8]
   - [if embedded in a practice] what do the GPs, nurses and other practice staff do?
   - How does this differ from what the ODNs do?
   - If not involved – what are your opinions on what GPs role should be in re-engagement?
9. What are some ways that your practice has used to communicate with patients with potential Hep C? [psychological capability, opportunity-social, 11]
   - Prompt for details: method of communication, how often? Does it depend on how/why lost to follow up and route of referral?
10. What are the challenges in patient communication? [capability-skills, social opportunity]
    - Prompts: do you feel confident in holding these conversations with patients? Is trust an issue? (11) Negative reactions from patients? Do you experience a lack of response or non-attendance of appointments?
    - What could be done differently?
11. Are there any different requirements for communicating with people lost to follow up, compared to new cases? [social opportunity]
12. How does primary care practice differ in patient-provider communication, to secondary care or other services, to your knowledge? [opportunity, psychological capability]
    - For instance, would a GP be better positioned to work with patients with complex needs – ?
13. What do you if you believe a patient is not ready for treatment or unlikely to complete their course of treatment? [motivation-professional role or identity, social opportunity 8]

General barriers and facilitators to both case-finding and re-engagement of lost to follow ups.

ESTABLISH RELEVANCE – OTHERWISE SKIP. The next questions are about the general benefits/downsides, motivations, and feelings about working on Hepatitis C.

14. Do you receive any incentives for any Hep C work? [motivation-reinforcement]
15. What encourages you to do your work? [motivation-beliefs about consequences]
16. Have you encountered any negative effects or outcomes? [motivation-reinforcement/beliefs about consequences]
    - Prompt: what discourages you?
17. Compared to everything else you do, how much of a priority is re-engaging Hepatitis C patients who are lost to follow up? [motivation-goals,8]
    - Prompt: Is it necessary, as a GP, to support re-engagement of HCV patients lost to follow up? [motivation-intention]
18. Would focusing on re-engagement of lost to follow up cases require a change to your role? or the roles of other practice staff? [motivation-social/professional role and identity]

19. To what extent are the supports and resources in place to enable you to be involved in hep c patient re-engagement? [opportunity-environmental context and resources, social interactions 8, 10]
    - If they don't have resources - what would be needed and for what purpose - e.g., for testing, assessing patients' suitability for treatment?
20. What are some strategies that GP practices could implement to monitor and improve on re-engaging patients? [psychological capability-behavioural regulation]
    - E.g., could they join up with drug and alcohol services, ODNs?

How optimistic are you that you can help support Hepatitis C case finding and re-engagement? [motivation-optimism]

21. Do you have any additional worries or concerns about working in Hepatitis C care? [motivation-emotions]

Final comments

22. Anything else you would like to add or reflect on?

Thank them, remind them they can get in touch with any further questions.

That there will be a report published and made available towards the end of the year.

ADDITIONAL NOTES FOR POTENTIAL DISCUSSION (OPTIONAL- IF they ask)

Some of what ODNs do includes:

- Offer treatment to people with positive PCR test and then review them afterwards (SVR testing)
- Keep people on a lost to follow up list and periodically contact them if they have refused treatment
- Some community testing nowadays – including the use of vans, home visits by community partners (such as the hepatitis c trust) – picking up new cases
- They receive some public health data (from UKHSA) to identify people who have moved to their area recently or have historical diagnosis with no record of being treated
- Some ODNs are working with GP practices to run the MSD PSI tool – to find people with risk factors (only happening is some parts of the UK)

What ODNs want the GPs to help with:

- Contacting patients they've been unable to make contact with – either by providing up to date contact details or making contact themselves
    - Either for initial testing, or adherence to treatment if they have been lost to follow up
- Offering testing locally if the patient doesn't want to travel to the hospital
- Informing patients of test results or helping them understand their results

- Letting the ODNs know if there's anything else known about the patient that makes it inappropriate to contact them at this time (e.g. they are receiving other treatment)
- Referring patients to the ODN if they have risk factors for Hep C
