# Supplementary material for: Barriers and facilitators to hepatitis C patient engagement: Interview study with general practitioner champions in England
Source: PLoS One. 2026 Jul 10;21(7):e0337867. doi: 10.1371/journal.pone.0337867 (PMC13353960; doi:10.1371/journal.pone.0337867)
Supplement: S2 File — (DOCX) [file pone.0337867.s002.docx]

**S2 Appendix – Full tables of themes**

Supp. Table 1. Full table of system level themes (extension of table 2 in the main document)

| All GPs or specific to GP champions | Theme | Barrier, Mixed, Facilitator | Number of Participants | Example quote | Related to patient identification, re-engagement or both? |
| --- | --- | --- | --- | --- | --- |
| All GPs | No financial incentives for Hepatitis C in primary care | Barrier | 7 | “it could be improved if there was some, you know, more stuff diffused down to practises, particularly if there's ten pence or so linked to it, you know and it because general practise is linked, you know, you might sound like they're all money grabbing, but it's basically a corner shop if you don't get paid for every little thing that you sell, you're going to go bankrupt and you won't be able to pay your staff.” (Participant 2) | Both |
|  | Pressures facing primary care | Barrier | 11 | “the issue of introducing more things for primary care to do is that. There's always lots of other things that people want to address. Diabetes and heart failure, and hepatitis c, smoking, alcohol. So I think it's how many ways you can slice up the work, the workload and GPs.” (Participant 12) | Both |
|  | Institutional practice changes related to prioritising hepatitis C | Barrier | 4 | “The NHS is, you know, so large it needs to take things, move so slowly.  …I mean, it's been internationally recognised for quite some time but NICE. So, the National Institute of Clinical Excellence, on their website which is called CKS, which is what a lot of doctors use, especially GPs for up-to-date information that is yet to be updated with what's been earmarked is Autumn 2024. So hopefully in the coming months and that may lead to then a change of coding within healthcare.  Which then may nudge along people who govern SNOMED^^[[1]](#footnote-2)^^” (Participant 4) | Both |
|  | Challenges to public trust in NHS/GPs | Barrier | 1 | “Obviously with the COVID pandemic and the kind of anti-primary care NHS rhetoric in the media, I'd say it's a harder job than it ever has been in terms of trust.” (Participant 12) | Both |
|  | Cyber security threats | Barrier | 2 | “I don't know if you know we've got a massive cyber-attack, so we've got no bloods right now….you know, people who've got diabetes, their checks are stopped right now. So Hep C is going to be probably on a 6 month hold in our area, to be honest.” (Participant 1) | Both |
|  | Previous treatments were associated with disengagement from care | Barrier | 5 | “we were still thinking about interferon treatment which had a very bad reputation. It was very difficult to use and then when Hep C treatment did come available, it was so expensive that we didn't even have the opportunity to refer our patients. So, we therefore were aware we had this cohort of patients, but then we weren't able to refer because it was so expensive and also the population, we were working with were very maybe difficult more to engage or to turn up. So those immediate patients were picked up and treated, but the patients that might have needed more, more adaptability to clinic times and flexibility weren't really treated, so we got all of the low-lying fruit and but not the harder ones to reach out.” (Participant 1) | Reengagement |
|  | Lack of standard procedure for re-engaging patients in primary care | Barrier | 8 | “it feels like there are kind of systems in place for secondary care in terms of where this person hasn't turned up twice, we can just discharge into primary care, but we've that's there's nowhere that we would then. There's no where that we can say, oh, once somebody's not coming twice and you discharge them into the world, I think there's a there's a sense that's the responsibility that is on you and for how often do you chase people up or how often do you engage them? So I think that's a very grey area.” (Participant 12) | Reengagement |
|  | Patient search tools and coding issues in GP practice systems | Mixed | 14 | “[The PSI^^[[2]](#footnote-3)^^ tool is] not a perfect search because it does pull in a number of risk factors and what that means is that in in real terms you get you can get depending on where you work very, very, very high numbers and actually. You know, a lot of those patients. Won't really need a test. If you look at. You look at it more and look at it in more detail, but I think it's a it's definitely a very good starting point. And yeah, we can refine it obviously.” (Participant 10) | Both |
|  | Patient care can be fragmented in referral to secondary care | Barrier | 2 | “obviously immediately directly when it's been confirmed that someone has been lost, to follow up and their hepatitis C positive is getting them into a into a clinic without the need for sort of another referral from say, GP. Which can obviously lead to fragmentation of their care.” (Participant 10) | Both |
|  | Named GP system in the NHS creates GP responsibility | Facilitator | 1 | “when the NHS introduced the named GP thing that was quite important for us because then I have, you know, I would end up with two and a half thousand patients and I would should be looking at their prescription and looking at them and where we were looking at you know if we looked at the QUOF^^[[3]](#footnote-4)^^ or something like that, there'll be this group of patients of mine. So, if they're not turning up, you know, that's my worry. Do you, do you see what I mean? I feel a bit more personally responsible.” (Participant 2) | Reengagement |
|  | Elimination funding from NHS England | Facilitator | 1 | [The hepatitis C elimination programme] “benefits massively from the kind of funding that's provided by NHS England and not entirely sure if that funding's, you know supported by the drug companies etcetera, but I think that that approach is clearly making a difference” (Participant 3) | Both |
|  | Digitisation of patient communication | Facilitator | 3 | “I think there's a real benefit to the kind of digitalization of care and you being able to use text messages and communicate much more freely.” (Participant 12) | Both |
| GP Champions | Challenges collaborating with primary care networks and regions | Barrier | 8 | “that took a lot of time to get the data protection policies in place, data sharing policies in place going through the governance, clinical governance, Caldicott guardians. But you know, so that did take a lot of meetings and a lot of documents, ultimately with patient care at the heart of it but you know, GP practices are very concerned with who has their patient data and how it gets shared and who contacts their patients. So, it really does need a lot of discussion with all system partners, but primarily that registered GP on how you know how they want us to work with their practice.” (Participant 14) | Both |
|  | Uncertain strategy for contacting large numbers of patients for testing | Barrier | 9 | “I know initially we've been thinking about sending kind of mass text messages out, but there've been some pushback against that being people, patients not liking to receive them. And the wording around those and people's sharing telephones. And is that text messages going to the right person? But if you make it too vague, nobody's going to reply. But if you look at too specific and you're at risk of breaking confidentiality.” (Participant 12) | Identification |
|  | Collaborating with the Operational Delivery Networks (ODNs) | Mixed | 10 | On collaborating with the ODN: “it's careful what you wish for isn't it? But there it's such a fantastic structure and it does, it's very responsive. So in some ways if people did know about it, I wonder whether there'll be more traffic because anything that makes a GPs job easier I think would be…” (Participant 12) | Both |
|  | Collaborating with drug and alcohol services | Mixed | 3 | “I have noticed a huge increase in [drug and alcohol services] ability to manage positive cases over the last couple of years so they would manage that a positive Hep C RNA result and referrals treatment without any involvement of the GP which is the way it should be. So yeah, they tend to manage their Hepatitis C patients independently of us.” (Participant 3) | Both |
|  | Collaborating with outreach services/charities/partners | Facilitator | 5 | “I've linked colleagues from the Hep C Trust with our local charities voluntary sector. In my boroughs, so in [areas] for the populations and ethnicities that we know are higher risk … we have a big deportation housing, in [Region] so we …they're doing a lot of work out in the community” (Participant 14) | Both |

## Table 2. Full table of provider level barriers and facilitators (extension of table 3 in the main report)

| All GPs or specific to GP Champions | Theme | Barrier, Mixed, Facilitator | Number of Participants | Example quote | Related to patient identification, reengagement or both? |
| --- | --- | --- | --- | --- | --- |
| Capability – physical and psychological skills of the person that make behaviour possible | | | | |  |
| All GPs | GPs have personal limitations | Barrier | 5 | “…we live in our own privileged bubbles as health professionals and we, but we perhaps lose sight of them [patients]…” (Participant 4) | Both |
|  | Knowledge and training in hepatitis C | Mixed | 14 | “I think a lot of the GPs then didn't realise how Hep C treatment has become. So, we have all these patients that we have. So, we have all these patients that we know in primary care are positive. However, we haven't really referred them on or done anything about them because we're GPs are still in that mindset.” (Participant 1) | Both |
|  | GP skills in managing conversations with patients about having Hepatitis C | Mixed | 8 | “We have all these patients and it's really really that are probably are eligible for Hep C tests, but we don't do enough risk assessments in primary care, and that's because it's a really difficult subject to sometimes bring up.” (Participant 1) | Both |
|  | Engaged in self-directed learning about hepatitis C | Facilitator | 3 | “yeah, and then, you know, a bit of reading here and there filling in the gaps.” (Participant 9) | Both |
| Opportunity – attributes of social or physical environment that make behaviour possible | | | | |  |
| All GPs | GPs lack capacity for additional hepatitis C work | Barrier | 10 | “but the only problem is because GPs are if they're overwhelmed, you know they can't be proactive because they're dealing with, you know, the clamour for appointments” (Participant 2) | Both |
|  | GPs are unable to contact some patients | Barrier | 8 | “some of them have clearly a little still registered with our practise, have probably left the UK. So, we've not had any involvement with them the last three or four years. So that's a bit of a known problem within general practise, a small proportion of your patients have probably left and we don't know anything about them.” (participant 3) | Both |
|  | Prevalence in practice area affects GP awareness or encounters with patients with hepatitis C | Mixed | 4 | “Interviewer: So, these are questions focusing on people that have been lost to follow up and it may or may not be relevant to your current work….  Participant 2: Well, I mean we haven't. You know, we're I'm in outer suburban [Region]… It's totally different from inner city [Region] and you can see that because it's there'll be far more cases. There'll be far more homeless people concentrated in the city centre” (Participant 2) | Both |
|  | Testing/treatment availability affects timing of bringing patients in | Barrier | 1 | “I envisage their availability and also the availability of the clinic, how quickly they can be seen because you want to keep the momentum when you contact someone, erm and and then I guess if they don't turn up on what do you do?” (Participant 5) | Both |
|  | Data sharing and digital resources | Mixed | 7 | “I think that there have been some people who love pop ups and some people hate them. But I think that that helped a bit because I think when you've hovered over it explained why that. … And but so I think [the digital standard blood panel] is good because it increases testing by maybe that doesn't help with awareness because people don't know what they're screening for. Yeah.” (Participant 12) | Both |
|  | Hepatitis C being curable improves patient conversations | Facilitator | 3 | “So I think Hepatitis C you've got more of a good news story. To offer the treatment and say that you're gonna be cured after. A subset of treatment, so that is something to a good tool to use.” (Participant 5) | Both |
|  | Hepatitis C training resources available for GPs | Mixed | 3 | “I did a module on the Royal College of GPs website hepatitis C… NHS Choices, …Yeah, but and there is a primary care tool kit from. … the hep C. C you later, group.” (Participant 9) | Both |
|  | Hepatitis testing resources | Mixed | 2 | “Yeah, we've got the we could test, yeah. In primary care, we can do the normal testing that you can send off to the lab. And then there's obviously the online.  Testing that patients can do at home as well. So we've got that, if that's what you mean from the point of view of resources as all the online stuff that you can print out for patients or send them via text message. So we've got all of that in our practises as well.” (Participant 7) | Both |
|  | GPs can re-engage patients by understanding and adapting to their needs | Facilitator | 9 | “it's having a flexible approach to bringing people in and making appointments is important.“ (Participant 12) | Reengagement |
|  | GPs holistic accessibility helps (re)engage patients | Facilitator | 9 | “we might have more of a relationship with people, there may be another reason that come here, they might come for medication, reviews or because they've got a sore throat and if you can tie it into that bring it up for a different reason while their there, then we have that advantage of the relationship and continuity.” (Participant 3) | Both |
| GP Champions | Learning and support from other GP Champions and multi-disciplinary healthcare professionals | Facilitator | 5 | “we have a do we have training like a training day once every six months?  …it's really useful. I think the most useful part is hearing is kind of the shared learning called case studies from other areas which is useful. And it's also nice to be in a room full of other GPs who are interested in the same thing.” (Participant 12) | Both |
|  | Capacity increases due to funded Champion role | Facilitator | 8 | “I guess probably for me it's [hepatitis c] relatively high up on my priority list, but that again, that is probably because I'm given the time and space to think about it and afforded that.” (Participant 3) | Both |
| Motivation – mental processes that energise and direct behaviour | | | | |  |
| All GPs | Hepatitis C ranking amongst other GP priorities | Mixed | 7 | “I don't think anybody's gonna do much chasing unless there's a driver for it. You know, so if they're in the quality outcomes framework and you know, they're finding those patients is critical to getting the reimbursement for the for that particular illness or whatever. Then there's going to be quite a lot of effort put into it. But if somebody otherwise you might send, you know, depending on how important the condition is, of course or how important the GP thinks the condition is, they might or might not chase them, but you know, if the people vanish, then they'll probably shrug their shoulders. They might send them a text message. Pretty rarely will they send them a letter because you know unless they think it's very important. But I don't think Hepatitis really comes into it that much because, yeah, it's just not high on GPs radar.” (Participant 14) | Both |
|  | Some GPs and other healthcare staff have negative attitudes towards patients | Barrier | 3 | “from my experience the only thing is that there is that stigma that may be associated. But I think I'm going to be honest, I think it's more of the professionals than the patients.” (Participant 14) | Both |
| GP Champions | Personal rewards and confidence improvements | Facilitator | 13 | “Yeah, it's really satisfying.  And I've learnt a lot about hepatitis C.  Which again, yeah, from a, I guess a clinical personal perspective. Very interesting. So I would say those are the main benefits and it's just it's not, it's just again it's variety in your working week. So it's something different.” (Participant 10) | Both |

Table 3. Full table of patient level themes (not included in main manuscript).

| Theme | Barrier, Mixed, Facilitator | Number of Participants | Example quote |
| --- | --- | --- | --- |
| Capability – physical and psychological skills of the person that make behaviour possible | | | |
| Language and comprehension | Barrier | 6 | “Language barriers can be an issue… but that can be a lack of understanding. And just lack of understanding sometimes there's probably more of a preference in [place] that they really just haven't understood, even though they speak English, they just haven't understood what you've said to them.” (Participant 7) |
| Lack of knowledge of new hepatitis C treatment | Barrier | 2 | “But often they [patients] weren't aware that there was any treatment for them, and they still don't. They still didn't think that there's treatment for them so.” (Participant 8) |
| Patients' psychological needs | Mixed | 3 | “that could be somebody who is who's got a serious mental illness. It might be somebody with dementia, …you know, people need advocacy quite a lot to find their way through the NHS if they if they have a disability, you know” (Participant 2) |
| Opportunity – attributes of social or physical environment that make behaviour possible | | | |
| Stigma or lack of referral to socially suitable centre/hospital | Barrier | 2 | “they know somebody at the clinic or that works at the hospital and they don't want to be seen going to attend an HIV clinic there, and so and they might, they didn't know that they could be seen somewhere else, and so they just disengage. “(Participant 5) |
| Patients are worried because they haven’t been listened to in the past | Barrier | 2 | “a lot of the time people feel a bit worried, but it's only because they haven't really been able to be listened to properly” (Participant 13) |
| Patients have competing social needs | Barrier | 4 | “And those patients, it's usually multifactorial. So yes, it's addressing this stigma… But a lot of the time for my patient group, it's social. It's what's going on down there. So, can we get in our social prescriber? Can we try and sort out their housing? Can we sort out their immigration status and get into a more stable place to be able to do this? So, her thing was ‘I'm homeless right now.’” (Participant 1) |
| Patients are receptive to normalising hepatitis C testing/testing promotions | Facilitator | 2 | “It's normalising it, it's it's making patients aware that it's something that we just offer as a standard test now because we know that people's risk is higher than they might think. And. And on the whole, patients are very receptive to that.” (Participant 10) |
| Patients benefit from peers and patient advocacy support | Facilitator | 5 | “Yes, yes, lived, lived experience. That's that makes a big impact when someone says I've had Hep C. Yes, I think they can help get the people and promote the DAA‘s so then we can get a cure.” (Participant 14) |
| Patients find GPs more accessible (than secondary care) | Facilitator | 2 | “I think the thing with primary care is that it is an accessible service. So, we have you know, so we are an accessible service that is free and available to everybody, and everyone can register for GP going on to secondary care treatment.” (Participant 1) |
| Motivation – mental processes that energise and direct behaviour | | | |
| Patients need high motivation to self-test | Barrier | 1 | “If someone doesn't really want to, you have to be quite motivated, I think, to order the tests, do it, prick your finger.” (Participant 9) |
| Patients choose whether to engage with care | Mixed | 10 | “the people are going to engage are going to engage. … we had a very similar patient who there was a safeguarding case because they just weren't engaging, and you know they just I got in contact with him and he sent me a letter, essentially a handwritten letter saying, well, actually, I seek all my care in the private sector. I spend most of my days abroad. I'm fine. Leave me alone.” (Participant 4) |
| Trust in healthcare providers | Mixed | 7 | “Then the challenge is trust in the healthcare profession, we find this with all sorts of things, you know, with hypertension, care with diabetes and things that people feel that they don't trust healthcare professionals and developing that relationship and also I guess if someone is cold calling you about Hepatitis C randomly.  You know who is this person and what have you?” (Participant 5) |

Table 4. Full table of GP interviewees suggestions for future hepatitis C care involving primary care.

| Suggestion | Intervention function/ policy category^[[4]](#footnote-5)^ | Number of participants | Example quote |
| --- | --- | --- | --- |
| Incentivise hepatitis C | Incentivisation / Fiscal measures | 7 | “GPs are incentivised for all their long-term conditions and I know Hepatitis C is not a long term condition because it's curable, but I think if we do want to achieve micro eliminations and things, we need to incentivise the GPs practices to do the work and to do the testing.… even the fact that there would be a financial incentive, it just puts it back on them, you know, puts it back on the agenda with the other conditions like COPD, diabetes, asthma. That kind of thing, yeah” (Participant 5) |
| Standardise regular testing/ offer as part of routine appointments and have GP reminder systems to maintain contact with people lost to follow up | Service provision | 8 | “We do need to think of like systematic ways of embedding it. So, it becomes like routine. So, you know like when we test for abnormal LFTs, Hep C just in there rather than us having to actively think do I need to add in a Hep C test just automatic in there wherever we move away from risk assessments in high prevalence areas to just automatically testing things like that. Then maintains the focus, but in a subdued way, you know, and it's embedded.” (Participant 1) |
| Increase the promotion of testing and treatment to patients | Education | 7 | “get information out there, you know on, you know, like we've obviously got a practise website, we have a Facebook page, we have, you know within this surgery waiting room, we could put posters that sort of thing. “(Participant 6) |
| Increase training and promotion to GPs and other practice staff | Education | 8 | “All clinical staff and to be honest non clinical staff, I mean you know really the gold standard that we should be aiming for is everyone being you know aware of how patients can access a test.” (Participant 10) |
| Include hepatitis C in inclusion health principles | Education | 2 | “I think potentially for GPs in the deprived area, it has to be a bit of a focus and has to fit in around kind of inclusion health principles, so inclusion health is the sort of care of people who have who have poor health outcomes from certain groups. So, people who experience in homelessness, asylum seekers people learn[ing] disabilities, people in in the prison service. So, I think if you're involved in in that group, it, it certainly should be a key part of your training, yeah.” (Participant 3) |
| Use the term out of care rather than lost to follow up | Guidelines, Communications | 1 | “Because lost to follow up its kind of I think it sort of is more of a negative connotation and actually, you know, is who's, you know, you put in the responsibility on that person when actually it's systemic. You know the reason why they might be out of care is because of things that are out of their control. You know, they don't have the money to come to clinic, the stigma. They're dealing. Yeah, they're dealing with other issues, so yeah, I think that's the reason.” (Participant id not included due to anonymity) |
| General views and recommendations on Hepatitis C elimination strategies  *~~.~~* | Service provision | 8 | “So, healthcare workers who don't have regular routine, they come, they come from other countries, but they've never been screened for bloodborne viruses, because it's not. You know, that should be. That should be part of the almost the contract of employment that everybody should, you know, it shouldn't even be down to GPs. It should be part of the occupational health that if you have somebody from another in at risk country, it should be your responsibility as an employer to make sure that this person has been tested. I mean, I think that's a big a big deficiency.” (Participant 2) |
| The need for GP Champions who can support other GPs | Service provision | 6 | “We're only going to be finding like each individual GP, practise is only going to be finding, say like two or three patients maybe.  So I still think that a lot of this needs to be done, not at a practise level, but at a systems level.” (Participant 8) |
| A joined-up approach is needed, including with secondary care/other services and/or other disease elimination strategies | Service provision | 6 | “having the joined-up approach that we are doing so much on HIV. Can we join up for Hep C and later TB and Hep B?” (Participant 1) |
| Create community primary care clinics / pharmacy DAA prescribers | Service provision | 5 | “I would like it that we could actually prescribe the drugs as well.  We can’t at the moment, we can only do it through the ODN, but I think actually having you know some- a few GPs with a special interest potentially being able to prescribe the drugs for the patients, I think it would make it, I know they can deliver them and things, but it's that engagement and getting them to then continue to take them and things which I think is really important.” (Participant 7) |

1. SNOMED CT: Systematized Nomenclature of Medicine Clinical Terms. [↑](#footnote-ref-2)
2. The [PSI: Patient Search Identification tool](https://www.emishealth.com/news-insights/emis-partners-with-nhs-england-and-msd), produced by MSD, searches for Hepatitis C risk factors in SNOMED codes in the EMIS patient record system. [↑](#footnote-ref-3)
3. QUOF: the Quality and Outcomes Framework is a voluntary reward/incentive programme for primary care practices in England. [↑](#footnote-ref-4)
4. See <https://assets.publishing.service.gov.uk/media/5fa537c7d3bf7f03b249aa12/UFG_National_Guide_v04.00__1___1_.pdf> - page 45, table 8. [↑](#footnote-ref-5)
