# Supplementary material for: Barriers and facilitators to hepatitis C patient engagement: Interview study with general practitioner champions in England
Source: PLoS One. 2026 Jul 10;21(7):e0337867. doi: 10.1371/journal.pone.0337867 (PMC13353960; doi:10.1371/journal.pone.0337867)
Supplement: Attachment — Submitted filename: Response to reviewers.docx [file pone.0337867.s003.docx]

Dear Dr. Stuart,

Thank you for submitting your manuscript to PLOS ONE. After careful consideration, we feel that it has merit but does not fully meet PLOS ONE’s publication criteria as it currently stands. Therefore, we invite you to submit a revised version of the manuscript that addresses the points raised during the review process.

==============================

Please note that we have only been able to secure a single reviewer to assess your manuscript. We are issuing a decision on your manuscript at this point to prevent further delays in the evaluation of your manuscript. Please be aware that the editor who handles your revised manuscript might find it necessary to invite additional reviewers to assess this work once the revised manuscript is submitted. However, we will aim to proceed on the basis of this single review if possible.

The reviewer raised a number of major concerns. They recommend that the manuscript clarify that most of the GPs are from London which may limit the generalizability of the study. They also made suggestions to improve the readability and structure of the manuscript.

Could you please carefully revise the manuscript to address all comments raised?

==============================

Please submit your revised manuscript by Mar 22 2026 11:59PM. If you will need more time than this to complete your revisions, please reply to this message or contact the journal office at [plosone@plos.org](mailto:plosone@plos.org). When you're ready to submit your revision, log on to <https://www.editorialmanager.com/pone/> and select the 'Submissions Needing Revision' folder to locate your manuscript file.

Please include the following items when submitting your revised manuscript:

- A letter that responds to each point raised by the academic editor and reviewer(s). You should upload this letter as a separate file labeled 'Response to Reviewers'.
- A marked-up copy of your manuscript that highlights changes made to the original version. You should upload this as a separate file labeled 'Revised Manuscript with Track Changes'.
- An unmarked version of your revised paper without tracked changes. You should upload this as a separate file labeled 'Manuscript'.

If you would like to make changes to your financial disclosure, please include your updated statement in your cover letter. Guidelines for resubmitting your figure files are available below the reviewer comments at the end of this letter.

If applicable, we recommend that you deposit your laboratory protocols in protocols.io to enhance the reproducibility of your results. Protocols.io assigns your protocol its own identifier (DOI) so that it can be cited independently in the future. For instructions see: [https://journals.plos.org/plosone/s/submission-guidelines#loc-laboratory-protocols](https://eur01.safelinks.protection.outlook.com/?url=https%3A%2F%2Ftrack.editorialmanager.com%2FCL0%2Fhttps%3A%252F%252Fjournals.plos.org%252Fplosone%252Fs%252Fsubmission-guidelines%2523loc-laboratory-protocols%2F1%2F010f019c31f5c2b8-2371f9ab-f31a-4693-8342-1202351f52ba-000000%2F5L71xm3fIMKoN36bhP-tw2GsAvPep_lWaD79RzhcrFI%3D249&data=05%7C02%7Cavelie.stuart%40ukhsa.gov.uk%7C66aa2edae42549a0b47008de6555889b%7Cee4e14994a354b2ead475f3cf9de8666%7C0%7C0%7C639059615506374562%7CUnknown%7CTWFpbGZsb3d8eyJFbXB0eU1hcGkiOnRydWUsIlYiOiIwLjAuMDAwMCIsIlAiOiJXaW4zMiIsIkFOIjoiTWFpbCIsIldUIjoyfQ%3D%3D%7C0%7C%7C%7C&sdata=JrmwnC%2FEGQrml%2Bhr%2FkIV9igVe0Iejchn6FK4frx0x0E%3D&reserved=0). Additionally, PLOS ONE offers an option for publishing peer-reviewed Lab Protocol articles, which describe protocols hosted on protocols.io. Read more information on sharing protocols at [https://plos.org/protocols?utm_medium=editorial-email&utm_source=authorletters&utm_campaign=protocols](https://eur01.safelinks.protection.outlook.com/?url=https%3A%2F%2Ftrack.editorialmanager.com%2FCL0%2Fhttps%3A%252F%252Fplos.org%252Fprotocols%253Futm_medium%3Deditorial-email%2526utm_source%3Dauthorletters%2526utm_campaign%3Dprotocols%2F1%2F010f019c31f5c2b8-2371f9ab-f31a-4693-8342-1202351f52ba-000000%2FyKkvVVMVoydewEbZ1j7sb6o3rZzn7UpXcFZIo9dMiDw%3D249&data=05%7C02%7Cavelie.stuart%40ukhsa.gov.uk%7C66aa2edae42549a0b47008de6555889b%7Cee4e14994a354b2ead475f3cf9de8666%7C0%7C0%7C639059615506408433%7CUnknown%7CTWFpbGZsb3d8eyJFbXB0eU1hcGkiOnRydWUsIlYiOiIwLjAuMDAwMCIsIlAiOiJXaW4zMiIsIkFOIjoiTWFpbCIsIldUIjoyfQ%3D%3D%7C0%7C%7C%7C&sdata=oNwKoZojgMiD%2B9lTM10spizBmP%2FVX%2Fw3hCRtdCF2dQI%3D&reserved=0).

We look forward to receiving your revised manuscript.

Kind regards,

Katherine Demi Kokkinias, Ph.D.
Staff Editor
PLOS One

Journal Requirements:

When submitting your revision, we need you to address these additional requirements.

1. Please ensure that your manuscript meets PLOS ONE's style requirements, including those for file naming. The PLOS ONE style templates can be found at

<https://journals.plos.org/plosone/s/file?id=wjVg/PLOSOne_formatting_sample_main_body.pdf> and

[https://journals.plos.org/plosone/s/file?id=ba62/PLOSOne_formatting_sample_title_authors_affiliations.pdf](https://eur01.safelinks.protection.outlook.com/?url=https%3A%2F%2Ftrack.editorialmanager.com%2FCL0%2Fhttps%3A%252F%252Fjournals.plos.org%252Fplosone%252Fs%252Ffile%253Fid%3Dba62%252FPLOSOne_formatting_sample_title_authors_affiliations.pdf%2F1%2F010f019c31f5c2b8-2371f9ab-f31a-4693-8342-1202351f52ba-000000%2FsRkWDVrB6NB5mYEiGoS5IxZz2hhVV18fHFwEMSwc32o%3D249&data=05%7C02%7Cavelie.stuart%40ukhsa.gov.uk%7C66aa2edae42549a0b47008de6555889b%7Cee4e14994a354b2ead475f3cf9de8666%7C0%7C0%7C639059615506428132%7CUnknown%7CTWFpbGZsb3d8eyJFbXB0eU1hcGkiOnRydWUsIlYiOiIwLjAuMDAwMCIsIlAiOiJXaW4zMiIsIkFOIjoiTWFpbCIsIldUIjoyfQ%3D%3D%7C0%7C%7C%7C&sdata=69RZFqLWJx8OLM79eICWTL3WbOkdEhSrcidBGnyv9dQ%3D&reserved=0).

Response: we have modified our manuscript to meet the style requirements.

2. Your ethics statement should only appear in the Methods section of your manuscript. If your ethics statement is written in any section besides the Methods, please delete it from any other section.

Response: We have removed the ethics statement from the additional section.

3. Please include captions for your Supporting Information files at the end of your manuscript, and update any in-text citations to match accordingly. Please see our Supporting Information guidelines for more information: <http://journals.plos.org/plosone/s/supporting-information>.

Response: We have added captions for our supporting information files and updated intext citations.

4. If the reviewer comments include a recommendation to cite specific previously published works, please review and evaluate these publications to determine whether they are relevant and should be cited. There is no requirement to cite these works unless the editor has indicated otherwise.

Response: Not applicable in our case.

[Note: HTML markup is below. Please do not edit.]

Reviewers' comments:

Reviewer's Responses to Questions

**Comments to the Author**

1. Is the manuscript technically sound, and do the data support the conclusions?

The manuscript must describe a technically sound piece of scientific research with data that supports the conclusions. Experiments must have been conducted rigorously, with appropriate controls, replication, and sample sizes. The conclusions must be drawn appropriately based on the data presented.

Reviewer #1: Yes

2. Has the statistical analysis been performed appropriately and rigorously?

Reviewer #1: Yes

3. Have the authors made all data underlying the findings in their manuscript fully available?

The [PLOS Data policy](https://eur01.safelinks.protection.outlook.com/?url=http%3A%2F%2Ftrack.editorialmanager.com%2FCL0%2Fhttp%3A%252F%252Fwww.plosone.org%252Fstatic%252Fpolicies.action%2523sharing%2F1%2F010f019c31f5c2b8-2371f9ab-f31a-4693-8342-1202351f52ba-000000%2FqxHSaysuCDPkXuWnsMlMAFmRC92V0fKIToEFFqxJiW0%3D249&data=05%7C02%7Cavelie.stuart%40ukhsa.gov.uk%7C66aa2edae42549a0b47008de6555889b%7Cee4e14994a354b2ead475f3cf9de8666%7C0%7C0%7C639059615506446434%7CUnknown%7CTWFpbGZsb3d8eyJFbXB0eU1hcGkiOnRydWUsIlYiOiIwLjAuMDAwMCIsIlAiOiJXaW4zMiIsIkFOIjoiTWFpbCIsIldUIjoyfQ%3D%3D%7C0%7C%7C%7C&sdata=ULHhjW03ROsoV40UtRpPROs82vOS11eg%2B%2BJPLMXtm%2Fo%3D&reserved=0) requires authors to make all data underlying the findings described in their manuscript fully available without restriction, with rare exception (please refer to the Data Availability Statement in the manuscript PDF file). The data should be provided as part of the manuscript or its supporting information, or deposited to a public repository. For example, in addition to summary statistics, the data points behind means, medians and variance measures should be available. If there are restrictions on publicly sharing data—e.g. participant privacy or use of data from a third party—those must be specified.

Reviewer #1: Yes

4. Is the manuscript presented in an intelligible fashion and written in standard English?

PLOS ONE does not copyedit accepted manuscripts, so the language in submitted articles must be clear, correct, and unambiguous. Any typographical or grammatical errors should be corrected at revision, so please note any specific errors here.

Reviewer #1: Yes

5. Review Comments to the Author

Please use the space provided to explain your answers to the questions above. You may also include additional comments for the author, including concerns about dual publication, research ethics, or publication ethics. (Please upload your review as an attachment if it exceeds 20,000 characters)

Reviewer #1: This study investigated the role of hepatitis C (HCV) General Practitioners (GPs) in identifying and re-engaging patients with HCV. Using semi-structured interviews and a dual analytical framework (a multi-level model and the COM-B behavioral framework), the authors identified barriers and facilitators to HCV care at the system level (e.g., NHS incentives, primary care pressures) and the provider level (e.g., GP knowledge, capacity, motivation) . They found that, while the GP Champion role is beneficial in terms of capacity, motivation, and collaboration, persistent system-level barriers (like lack of financial incentives and fragmented processes) hinder wider success. The study attempted to address a critical public health gap- finding “lost” patients to achieve HCV elimination goal. However, this is a purely qualitative study, lack of quantitative data is the key limitation.

Additionally, the concerns listed below must be addressed.
1. The participant sample is heavily weighted toward London-based GPs (10 of 14), which may limit the representativeness of the findings across England. This should be clearly acknowledged in the limitations section.

Response: we have specified in the Strengths and limitations section (p. 27, ln. 963) that “the findings were specific to the NHS and England, with most GPs interviewed being based in London. Therefore, the findings may not all be applicable in other regions of the UK or internationally”

We also clarified in the introduction (p. 6, ln 240) that GP Champions were initially employed in London, before the programme was extended nationally.

2. The manuscript title appears to overstate the generalizability of the findings. As the participants were predominantly hepatitis C GP Champions (12 of 14), rather than general GPs, the title should be revised to more accurately reflect the study population.

Response: we have renamed the manuscript, specifying GP Champions as the primary interviewees: “Barriers and facilitators to hepatitis C patient engagement: interview study with General Practitioner Champions in England”
3. The Introduction section is somewhat lengthy. A light condensation would improve clarity and focus. In particular, the discussion of international decentralization efforts could be slightly shortened.

Response: We have shorted the introduction section, in particular the discussion of decentralization of treatment. Changes are highlighted using track changes in the manuscript.

4. The Introduction would benefit from a more explicit and concise statement of the research gap that this study aims to address.

Response: we have modified the study aims statement in the ‘current study’ section (p. 6 ln 129) to explicitly state how the GP Champion programme provided a novel opportunity to study barriers/facilitators to hepatitis C care:

“This study employs expert interviews [30]. An NHS England-commissioned hepatitis C GP Champion programme is operating with (at the time) 50 Champions employed initially in London, and then extended nationally. This provides us an opportunity to investigate GPs experiences of the barriers and facilitators to identifying, (re)engaging, and potentially treating patients living with hepatitis C via primary care in England”

5. The phrasing in lines 47–50 is ambiguous, particularly regarding the relationship between progress made and the remaining disease burden. Clarification would improve readability.

Response: We have rephrased this sentence to the following: “In England there has been a decrease of 56.7% of people living with chronic hepatitis C since 2015, with a remaining estimated 55,900 people who are living with hepatitis C”

6. The Conclusion section is overly long and reads as descriptive rather than synthetic. Repeated use of the phrase “they reported” gives the section a results-like tone. The authors may consider tightening this section and emphasizing a clearer, more focused final takeaway.

Response: We have reduced the length of the conclusions section as well as removed the phrase “they reported”. The conclusion now consists of the following shorter paragraph (p. 28):

“This study explored barriers and facilitators faced by GPs who are engaged in identifying and (re)engaging patients living with hepatitis C. GP Champions experiences with receiving funding and support from other GP Champions enable them to overcome some of the barriers facing GPs, particularly capacity and knowledge limitations. Additionally, Champions experienced professional benefits such as improved confidence in working with patients with hepatitis C and finding the work personally rewarding. However, new challenges were identified, and persistent challenges that have not yet been addressed. These included: difficulties coordinating efforts to find and (re)engage patients across regions; searching patient records and the complexity of digital reminders to (re)offer patients hepatitis C tests; and the need for a holistic and flexible approach to meet patient needs. Suggestions for future work towards hepatitis C elimination entailed joining up blood-borne virus elimination campaigns and services, further incentives and training for hepatitis C, and community multi-disciplinary clinics to provide holistic care including DAA prescriptions.”

Thank you for these comments that have enabled us to improve our manuscript.
